# Pharmacogenomics and Herb-Drug Interactions: Merge of Future and Tradition

**DOI:** 10.1155/2015/321091

**Published:** 2015-03-02

**Authors:** Mou-Ze Liu, Yue-Li Zhang, Mei-Zi Zeng, Fa-Zhong He, Zhi-Ying Luo, Jian-Quan Luo, Jia-Gen Wen, Xiao-Ping Chen, Hong-Hao Zhou, Wei Zhang

**Affiliations:** ^1^Department of Clinical Pharmacology, Xiangya Hospital, Central South University, Changsha 410008, China; ^2^Hunan Key Laboratory of Pharmacogenetics, Institute of Clinical Pharmacology, Central South University, Changsha 410078, China

## Abstract

The worldwide using of herb products and the increasing potential herb-drug interaction issue has raised enthusiasm on discovering the underlying mechanisms. Previous review indicated that the interactions may be mediated by metabolism enzymes and transporters in pharmacokinetic pathways. On the other hand, an increasing number of studies found that genetic variations showed some influence on herb-drug interaction effects whereas these genetic factors did not draw much attention in history. We highlight that pharmacogenomics may involve the pharmacokinetic or pharmacodynamic pathways to affect herb-drug interaction. We are here to make an updated review focused on some common herb-drug interactions in association with genetic variations, with the aim to help safe use of herbal medicines in different individuals in the clinic.

## 1. Introduction

Herbal medicinal products (HMPs) belong to a main part of complementary and alternative medicine (CAM). The National Center for Complementary and Alternative Medicine (NCCAM) has defined CAM as those health care practices not currently considered an integral part of conventional medicine [1]. During the past two decades, the world has witnessed an increased rate of conventional medicines integrated with CAM for treatment. It was estimated that in 1990 about one-third of the US population used unconventional therapies and the expenditures on this amount to $13.7 billion [2], whereas in 2007 approximately 38 percent of American adults (83 million) are using some form of CAM for their health, and the expenditures reached $34 billion [1]. Correspondingly the funding for NCCAM has reached $124.125 million in 2013, much more than that was in 1998 ($19.5 million) when it was established [1]. In eastern Asia, countries that have a tradition use of oriental herb medicine for centuries, the prevalence of CAM use is much higher. In Japan, the use of CAM in the general population had been reported to be 76% [3]. In China, 93.4% of cancer patients reported having used CAM in 2009-2010 [4]. In South Korean population reported a range from 29% to 83% [5].

The prevalence of HMPs and other forms of CAM for treatment have its reasons. Conventional medicines are relatively more expensive, more difficult, and inconvenience of access and sometimes more likely to get side effects, toxicities, and incomplete efficacy. CAM, such as traditional Chinese medicine (TCM), though empirical, has been used for centuries and proved to be effective. In developing countries like China patients, especially those in rural areas, are more likely to choose HMPs as main medicine for treatment. In developed countries or areas like Japan, the increased use of CAM has promoted the government approval of 148 traditional Japanese (or Chinese) herbal medicines listed on the “National Health Insurance Drug Tariff” [6]. Western countries, however, in most cases use herb prescriptions as dietary supplements or as part of a traditional medicine, and this trend is still rising. This increased the chance of concurrently use of herbs and conventional drugs. Similar to drug-drug interactions, the multicomponent herbs can exert their effects on pharmacokinetics and consequently lead to herb-drug interactions (HDIs) [7]. Understanding the exact mechanism of each extract of an herb that affects drug's absorption, distribution, metabolism, and excretion (ADME) properties is incomplete at the moment. A review of previous studies indicated that pharmacogenetic factors may help to explain the HDIs effect and give us instructions for rational drug administration.

## 2. Herb-Drug Interactions

The incidences of herb products to affect drug responses maybe more often in the clinic than expected for it is more easy for patients to conduct self-administration without informing their health care providers. Some of the interactions may have a beneficial effect by increasing drug efficacy or diminishing potential side effects. For example, combined therapy of garlic (250 mg/kg) with captopril demonstrated higher synergistic action with respect to fall in blood pressure and ACE inhibition [8]; patients received silymarin (140 mg three times daily) in combination with conventional desferrioxamine therapy showed beneficial effects on thalassemia patients [9]. However, more often the potential side-effect and outcome influence may be brought about by combination use of HMPs and conventional drugs, and these circumstances are always difficult to predict. Previous studies identified various mechanisms of pharmacokinetic HDIs, mainly mediated by drug-metabolizing enzymes and transporters [10]. For example, St. John's wort (*Hypericum perforatum*) significantly reduced the area under the plasma concentration-time curve (AUC) and blood concentrations of cyclosporine, midazolam, tacrolimus, amitriptyline, digoxin, indinavir, warfarin, phenprocoumon, and theophylline. Most of them are substrates of cytochrome P450s (CYPs) and/or P-glycoprotein (P-gp) [11]. An important fact is that most of these affected drugs have very narrow therapeutic indices. So, adverse drug reactions, toxicities, and treatment failure are more likely to occur when they are integrated with herbs. To date an increasing number of studies in evaluating HDIs have been reported [11].

CYP enzymes can be induced by various herbs. It was reported that Danshen-Gegen formula (DGF) could induce the liver phase I metabolism of warfarin, especially CYP1A1 and CYP2B1, as a result, it increased the intestinal absorption of warfarin by >30% and decreased its plasma protein binding by >11.6% [12].

A clinic case reported that preadministration of St. John's wort significantly decreased stable plasma level of clozapine from 0.46–0.57 mg/L to 0.19 mg/L, after discontinuation of St. John's wort for a month, the clozapine concentration restored to normal [13]. As the author discussed, St. John's wort may pharmacokinetically interact with clozapine through inducing a number of CYP450 enzymes such as CYP3A4, CYP1A2, CYP2C9, and CYP2C19, which are responsible for clozapine metabolism. In another case it is reported that* Ginkgo biloba* can negatively influence the effect of antiretroviral drug Efavirenz (EFV) in a HIV infected male patient. This may due to the inducing effect of* Ginkgo biloba* on the EFV metabolism enzymes CYP2B6 and CYP3A4 [14].

Recently, an in vivo study showed that coadministration of a single dose of baicalin, a Chinese medicine isolated from* Scutellaria baicalensis*, resulted in a dose-dependent decrease of midazolam in its clearance (CL) from 25% to 34%, while it increased in AUC_0–*∞*_ from 47% to 53%. Pretreatment of baicalin also reduced midazolam CL by 43%, with an increase in AUC_0–*∞*_ by 87%. Meanwhile, multiple doses of baicalin decreased the expression of hepatic CYP3A2 by approximately 58%. This indicated that baicalin induced changes in the pharmacokinetics of midazolam may be due to its inhibition of CYP3A in the liver [15]. There is a letter that analyzed a number of separated studies and reported that a well-known Chinese medicine ginseng may have interaction with anticoagulant warfarin by reducing warfarin's plasma levels and anticoagulant effect in ischemic stroke patients [16]. However, the exact mechanism of ginseng-warfarin interaction is unknown and needs to be thoroughly investigated.

Most of study reported that HDIs mechanisms are associated with pharmacokinetics and pharmacodynamics related genes, mainly including CYP450 enzymes and/or P-glycoprotein (P-gp) [11, 17] and also UDP-glucuronosyltransferases (UGTs) [18]. For more details we can refer to the reviews [11, 19, 20]. Some studies found that herb induced expression changes of genes may have some specificity; for instance, it was reported that coadministration of* Marsdenia tenacissima* extract (MTE) with gefitinib significantly decreased the in vitro intrinsic clearance (Clint) of gefitinib by 2.6- and 4.0-fold for CYP2D6 and CYP3A4, respectively, but did not affect other CYP450s [21]; another study reported that hyperoside could selectively inhibit CYP2D6 activity in a dose dependent manner and might cause herb-drug interactions when coadministrated with CYP2D substrates [22].

However, whole herbs like garlic, St. John's wort, Gingko, and so forth are complex compounds. Various confounding factors driving the pharmacokinetic profiles of them are considered to be a crucial factor when evaluating HDIs. In these cases we should make sure the confounding factors are at the same levels in the research situations. While evaluating the pharmacokinetic synergy effects of different ingredients in a whole herb on HDIs remains a big challenge so far. Previous studies have made us believe that the genetic involvement in the pharmacokinetic pathways may help us to understand this effect. Recent pharmacogenomics studies in the following and cases listed in Tables 1 and 2 of these pharmacokinetics and pharmacodynamics related genes may help explain the individual differences in HDIs.

## 3. Pharmacogenomics in HDIs Mechanisms

Genetic polymorphisms have long been studied and believed to involve pharmacokinetic and pharmacodynamic pathways that cause individual difference in drug responses [47]. Herbal medicines are actually a combination of potentially biologically active compounds possessing various inherent pharmacological activities. The metabolism of these compounds usually occurs by the same mechanisms as that of drugs. Pharmacokinetic interactions mediated by drug-metabolizing enzymes or transporters are involved in many herb-drug interactions [48], and as the concentrations of the herbal extract and the drug may determine the degree of DHI, polymorphisms in the drug-metabolizing enzyme genes (listed in Table 1) and transporter genes (listed in Table 2) that alter the systemic exposure to the substrate drugs or active components of herbs may affect the risk of interaction [10, 48, 49]. On the other hand, drug metabolism or transport has alternative pathways. When poor metabolizer genotype or inhibition of an enzyme may lead to the drug metabolized by another pathway enzymes, which may be more sensitive to their native substrates, then it may cause the competition metabolism of drugs or herbs and eventually HDIs difference. So conducting pharmacogenomics studies on HDIs may help to illuminate the fundamental mechanisms underlying the HDIs. In the following, we reviewed pharmacogenomics studies, mainly from clinic study, on some important herb products that have been reported to have HDIs.

### 3.1. St. John's Wort

St. John's wort is an herb most commonly used for depression and conditions that sometimes go along with depression such as anxiety, tiredness, loss of appetite, and trouble in sleeping. There is some strong scientific evidence that it is effective for mild to moderate depression. Currently it is displayed that 95 drugs (296 brand and generic names) are known to have a major interaction with St. John's wort. After administration of St John's wort, the AUC_0–*∞*_ of nifedipine and dehydronifedipine decreased by 42.4% and 20.2% in PXR H1/H2 genotype and 47.9 and 33.0% in H2/H2 genotypes, whereas for the H1/H1 the AUC_0–*∞*_ of nifedipine decreased by 29.0%, but the AUC_0–*∞*_ of dehydronifedipine increased by 106.7% [30]. St. John's wort treatment significantly increased phenytoin clearance in CYP2C19 EMs (^*^2, ^*^3) but not in PMs and decreased the plasma concentrations of omeprazole in a CYP2C19 genotype-dependent manner [33]. Subjects harbouring the ABCB1 haplotype comprising 1236C>T, 2677G>T/A, and 3435C>T polymorphisms had lower intestinal MDR1 mRNA levels and showed an attenuated inductive response to St. John's wort as assessed by talinolol disposition [45]. The AUC of voriconazole was decreased by 59% with St. John's wort treatment, with a 144% increase in oral clearance of voriconazole. The baseline apparent oral clearance of voriconazole and the absolute increase in apparent oral clearance were smaller in CYP2C19^*^2 carriers than those with CYP2C19^*^1/^*^1 genotype [32].

### 3.2. Ginkgo

Ginkgo is often used for memory disorders including Alzheimer's disease. It is also used for conditions that seem to be due to reduced blood flow in the brain, especially in older people. These conditions include memory loss, headache, ringing in the ears, vertigo, difficulty concentrating, mood disturbances, and hearing disorders. Ginkgo enhanced omeprazole hydroxylation in a CYP2C19 genotype-dependent manner. The decrease was greater in CYP2C19 PMs (^*^2, ^*^3) than in EMs [25].

### 3.3. Baicalin

Baicalin is a flavone glucuronide purified from the medical plant* Radix scutellariae* through uridine diphosphate glucuronidation. Nowadays, baicalin has begun to be used in bilirubin lowering therapy, both prescribed and over the counter, in China. The mean changes in AUC ratio of bupropion was lower for subjects with CYP2B6^*^6/^*^6 genotype compared with those with CYP2B6^*^1/^*^1 genotype following baicalin use, indicating baicalin-caused induction of CYP2B6-catalyzed bupropion hydroxylation. And administration of baicalin decreased the AUC_0–*∞*_ of rosuvastatin by about 42%, 24%, and 1.8% in SLCO1B1 ^*^1b^*^1b, ^*^1b^*^15, and ^*^15^*^15 carriers, respectively [23].

### 3.4. Garlic

Garlic is widely used around the world for its pungent flavor as a seasoning or condiment. There is some scientific evidence that garlic can lower high cholesterol after a few months of treatment. Garlic seems to also lower blood pressure in people with high blood pressure and possibly slow “hardening of the arteries.” There is also some evidence that eating garlic might reduce the chance of developing some cancers such as cancer of the colon and possibly stomach cancer and prostate cancer. Coadministration of garlic did not significantly alter warfarin pharmacokinetics or pharmacodynamics. However, subjects with the VKORC1 wild-type genotype showed an increase in the S-warfarin EC50 when warfarin was administered with garlic [50].

### 3.5. Grapefruit Juice

Grapefruit juice, and grapefruit in general, is a potent inhibitor of the cytochrome P450 CYP3A4 enzyme, which can affect the metabolism of a variety of drugs, increasing their bioavailability. In some cases, this can lead to a fatal interaction with drugs like astemizole or terfenadine. Grapefruit juice treatment significantly increased total AUC of lansoprazole in CYP2C19 PMs (^*^2, ^*^3), and the total AUC of lansoprazole sulfonic/lansoprazole was significantly decreased in CYP2C19 homozygous EMs (^*^1/^*^1) [26]. Homozygous wild types of ABCB1 3435C>T but not the other genotypes showed a significant decrease in the active metabolite carebastine urinary excretion after grapefruit juice [43].

### 3.6. Cranberry Juice

Cranberry juice is the juice of the cranberry. Cranberry juice contains phytochemicals, which may help prevent cancer and cardiovascular disease. Cranberry juice is high in oxalate and has been suggested to increase the risk for developing kidney stones, although more recent studies have indicated it may lower the risk. Cranberry significantly increased the area under the INR-time curve by 30% when administered with warfarin without altering pharmacokinetics or plasma protein binding of S- or R-warfarin, and this effect is dependent on VKORC11173T>C polymorphism. Subjects with CT and TT genotypes coadministered with cranberry juice extract significantly reduced S-warfarin EC50 (concentration of S-warfarin that produces 50% inhibition of prothrombin complex activity) by 22% and 11%, respectively [50]. This case gives us an example of pharmacodynamic pathway gene polymorphisms that can be involved in the herb-drug interaction.

### 3.7. Tianqi Jiangtang

Tianqi Jiangtang is an herb widely used for diabetes treatment in China. Tianqi Jiangtang consists of 10 Chinese herbal medicines, namely, Radix Astragali, Radix Trichosanthis, Fructus Ligustri Lucidi, Caulis Dendrobii, Radix Ginseng, Cortex Lycii Radicis bone, Rhizoma Coptidis, Asiatic Cornelian cherry fruit, Ecliptae Herba, and Chinese gall. Many of these herbal medicines are correlated with diabetes-related parameters. For example, Rhizoma Coptidis and astragalin in Radix Astragali reduce glucose, similar to Diformin. Berberine in Rhizoma Coptidis improves some glycemic parameters. Ginsenoside Re in Radix Ginseng has significant antihyperglycemic effects. The iridocytes of* Cornus officinalis* in Asiatic Cornelian cherry fruit prevent diabetic vascular complications. Only one major effective component of Tianqi Jiangtang has been identified, namely, berberine hydrochloride (C_20_H_18_ClNO_4_), which has been successfully employed in antidiabetic treatments.

A total of 194 impaired glucose tolerance (IGT) subjects treated with Tianqi Jiangtang for 12 months were genotyped for 184 mutations in 34 genes involved in drug metabolism or transportation. The rs1142345 (A>G) SNP in the thiopurine S-methyltransferase (TPMT) gene was significantly associated with the hypoglycemic effect of the drug (*P* = 0.001, FDR *P* = 0.043). The “G” allele frequencies of rs1142345 in the healthy (subjects reverted from IGT to normal glucose tolerance), maintenance (subjects still had IGT), and deterioration (subjects progressed from IGT to T2D) groups were 0.094, 0.214, and 0.542, respectively. Rs1142345 was also significantly associated with the hypoglycemic effect of the drug between the healthy and maintenance groups (*P* = 0.027, OR = 4.828) and between the healthy and deterioration groups (*P* = 0.001, OR = 7.811). Therefore, rs1142345 was associated with the clinical effect of traditional hypoglycemic herbs. This is the first study to utilize the ADME gene chip in the pharmacogenetic study of traditional herbs [35].

## 4. Conclusion and Future Perspectives

Due to the limitations of conventional drugs, the use of herbal medicinal products (HMPs) as a CAM integrated with drugs for treatment has been increasing, this eventually will contribute to a rising incidence of herb-drug interactions. However, because of variability in herbal product composition, uncertainty of the causative constituents, and often scant knowledge of causative constituent pharmacokinetics, the mechanisms underlie herb-drug interactions remain an understudied area, and evaluation of herbal product interaction liability is still a big challenge [19]. Studies showed that these HDIs are mediated mainly by metabolism enzymes and transporters in the pharmacokinetic pathways. Polymorphisms in these pharmacokinetic pathways related genes can contribute to the individual difference of HDIs effect. We make an updated review with respect to pharmacogenomics related HDIs on some important herb products and call for a vigilance of using these drug for therapy when the patient is taking medication with narrow therapeutic indices.

Currently, there is still a lack of effective method to predict HDIs in the clinic; the main obstacles are laid by the multiconstituents of an herb. In the near future years, scientist in this area should focus on identifying individual constituents from herbal products, characterizing pharmacokinetics and pharmacodynamics of each individual constituents, discovering the fundamental genetic bases that eventually facilitate prospective identification of herb-drug interactions. Here we reviewed a definite involvement of pharmacogenomics in HDIs; some other mechanism, such as epigenetic regulations, that is, a recent hotspot area, is also supposed to take part in it.

The National Institute of Health (NIH) and similar organizations in other countries have increased the financial support for scientific investigation on safety use of herb medicines. This trend will promote the herb medicines more normalization as that of drugs, and the progress may promote finding new effective drugs from extracts of herbs. With respect to the wide and increasing use of herb drugs and their benefits, and with the progress in pharmacogenomics and pharm-chemistry, we are still optimistic on this area.

## Figures and Tables

**Table 1:** Metabolic enzyme gene polymorphisms and HDIs.

Herbal medicines	Drugs	Genes	Polymorphisms	Pharmacogenomics in HDIs effects	References
Baicalin	Bupropion	CYP2B6	^*^1/^*^1, ^*^1/^*^6, ^*^6/^*^6	The AUC ratio of hydroxybupropion to bupropion tended to be more pronounced lower in ^*^6/^*^6 compared with ^*^1/^*^1 genotype patients (5.3 versus 8.0) after baicalin treatment.	[23]

Echinacea	Dextromethorphan	CYP2D6	Extensive metabolizers Poor metabolizer	Echinacea dosing reduced the oral clearance of dextromethorphan by 28% and increased AUC by 42% in the CYP2D6 poor metabolizers, while extensive metabolizers were not affected.	[24]

*Ginkgo biloba *	Omeprazole	CYP2C19	EMs (^*^1/^*^1, ^*^1/^*^2, and ^*^1/^*^3); PMs (^*^2/^*^2 and ^*^2/^*^3)	After *G. biloba*, the mean decreases in omeprazole AUC_0–*∞*_ were 41.5%, 27.2%, and 40.4% in the homozygous Ems and heterozygous EMs and PMs, whereas the decreases in omeprazole sulfoneomeprazole sulfone AUC_0–*∞*_ were 41.2%, 36.0%, and 36.0%, respectively.	[25]

Grapefruit juice (GFJ)	Lansoprazole	CYP2C19	^*^1/^*^1, ^*^1/^*^2, ^*^1/^*^3, ^*^2/^*^2, and ^*^2/^*^3	GFJ treatment significantly increased total AUC of lansoprazole (26661 ± 7407 versus 34487 ± 10850 ng·h/mL) in ^*^2/^*^2 and ^*^2/^*^3 subjects, whereas it was significantly decreased (0.07 ± 0.05 versus 0.04 ± 0.05) in ^*^1/^*^1 genotype carries.	[26]

Grapefruit juice	Lansoprazole	CYP2C19	homEMs (^*^1/^*^1) hetEMs (^*^1/^*^2 and ^*^1/^*^3); PMs (^*^2/^*^2 and ^*^2/^*^3)	The mean plasma concentrations of lansoprazole were not increased by GFJ, whereas GFJ slightly prolonged the *t*_max⁡_ in the three different CYP2C19 genotype groups.	[27]

Liu Wei Di Huang Wan (LDW)	Omeprazole, dextromethorphan hydrobromide, and midazolam	CYP2C19, CYP2D6, CYP3A4	CYP2C19^*^1/^*^1 CYP2C19^*^2/^*^2	LDW is unlikely to cause pharmacokinetic interaction when it is combined with other medications predominantly metabolized by CYP2C19, CYP2D6, and CYP3A4 enzymes.	[28]

Silymarin	Losartan	CYP2C9	^*^1/^*^1 and ^*^1/^*^3	The metabolic ratio of losartan (ratio of AUC_0–*∞*_ of E-3174 to AUC_0–*∞*_ of losartan) after a 14-day treatment with silymarin decreased significantly higher in individuals with the CYP2C9^*^1/^*^1 genotype (48.78 ± 25.85%) than the CYP2C9^*^1/^*^3 genotype (20.09 ± 16.87%).	[29]

St. John's wort	Nifedipine	PXR CYP3A4	PXR haplotypes H1 and H2	Administration of St. John's wort induces higher metabolic activity of CYP3A4 in H1/H1 than in H1/H2 and H2/H2 subjects, with the AUC_0–*∞*_ of Nifedipine decreased by 42.4% (H1/H2), 47.9% (H2/H2), and 29.0% (H1/H1), whereas that of dehydronifedipine increased by 20.2%, 33.0%, and 106.7%.	[30]

St. John's wort	Gliclazide	CYP2C9	^*^1/^*^1 and ^*^1/^*^2 or ^*^2/^*^2 and ^*^1/^*^3	Treatment with St. John's wort significantly increases the apparent clearance of gliclazide by 50%. For CYP2C9^*^2 allele carriers, the increase is slightly lower.	[31]

St. John's wort	Voriconazole	CYP2C19	^*^1/^*^1, ^*^1/^*^2, and ^*^2/^*^2	The AUC of voriconazole was increased by 22% the first day and decreased by 59% after 15 days, with a 144% increase (carriers of 1 or 2 deficient CYP2C19^*^2 alleles were smaller than wild-type carries) in its oral clearance (CL/F) after St. John's wort administration.	[32]

St. John's wort	Omeprazole	CYP3A4 CYP2C19	^*^1/^*^1, ^*^2/^*^2, and ^*^2/^*^3	St. John's wort can induce CYP3A4 and increase higher CYP2C19 activity in wild-type than poor metabolizers (^*^2/^*^2 or ^*^2/^*^3), leading to increased metabolites of omeprazole in genotype-dependent manner.	[33]

St. John's wort	Mephenytoin and caffeine	CYP2C19	^*^1/^*^1, ^*^2/^*^2, and ^*^2/^*^3	St. John's wort treatment significantly increased CYP2C19 activity in ^*^1/^*^1 subjects, with mephenytoin metabolites excretion raised by 151.5% ± 91.9%, which is in contrast to ^*^2/^*^2 and ^*^2/^*^3 individuals.	[34]

Tianqi Jiangtang	Unknown	TPMT	rs1142345	The effective ratio of subjects with homozygotes (AA) of the wild-type allele of TPMTrs1142345 was 2.8 times higher than that of subjects with TPMT heterozygotes (AG).	[35]

Yin zhi huang (YZH)	Omeprazole	CYP3A4 CYP2C19	CYP2C19 (^*^1/^*^1, ^*^1/^*^2, ^*^1/^*^3, and ^*^2/^*^2)	YZH induces CYP3A4 and CYP2C19 metabolism of omeprazole with the decrease of the AUC_0–*∞*_ ratio of omeprazole/5-hydroxyomprazole in CYP2C19^*^1/^*^1 and CYP2C19^*^1/^*^2 or ^*^3 greater than in CYP2C19^*^2/^*^2.	[36]

AUC: concentration-time curve; EMs: extensive metabolizer; PM: poor metabolizer.

**Table 2:** Transporter gene polymorphisms and HDIs.

Herbal medicines	Drugs	Genes	Polymorphisms	Pharmacogenomics in HDIs effects	References
Apple juice	Fexofenadine	SLCO2B1	c.1457C>T	Administering with apple juice decreased the fexofenadine AUC compared with control (1342 ± 519 versus 284 ± 79.2 ng·h/mL). The apple juice induced decrease in fexofenadine AUC was significantly lower in subjects carrying the c.[1457C>T] allele.	[37]

Baicalin	Rosuvastatin	OATP1B1	^*^1b/^*^1b; ^*^1b/^*^15; ^*^15/^*^15	After baicalin treatment, the AUC_0–72_ and AUC_0–∞_ of rosuvastatin were decreased according to OATP1B1 haplotype ^*^1b/^*^1b (47.0 ± 11.0% and 41.9 ± 7.19%), ^*^1b/^*^15 (21.0 ± 20.6% and 23.9 ± 8.66%), and ^*^15/^*^15 (9.20 ± 11.6% and 1.76 ± 4.89%), respectively.	[38]

Berberine Evodiamine	Fluoxetine and Sertraline may be affected	Serotonin transporter (5-HTT)	S, XS11, XL17, XL18 alleles and A>G (rs25531)	When tested against the S, XS11, LG, LA, XL17, and XL18 alleles, 100 mM berberine increased 5-HTT promoter activities by 67%, 128.7%, 106.9%, 100.4%, 26.2%, and 82%, 2 mM evodiamine increased 5-HTT promoter activities by 216.7%, 81.6%, 305.6%, 181.5%, 175.3%, and 102.2%, respectively.	[39]

Citrus juice	Montelukast	SLCO2B1	c.935G>A (rs12422149)	When coingested with orange juice, the AUC_0–∞_ of montelukast detected with a significant reduction in G/G homozygotes compared with control (2010 ± 650 versus 2560 ± 900 ng·h/mL).	[40]

*D.dasycarpus P. cocos*, *R.verniciflua* stokes	Digoxin and daunorubicin	MDR1	2677G/T/A (rs2032582)	Digoxin effluxes were significantly decreased in MDR1 gene variants of 2677T/893Ser with the treatment of *P. cocos* and of 2677G/893Ala and 2677T/893Ser with the treatment of *D. dasycarpus. *	[41]

Radix Astragali (RA)	Fexofenadine	ABCB1	C3435T	*T*_1/2_ of fexofenadine in ABCB1 3435T mutation allele carriers was longer compared to ABCB1 3435CC carriers (4.43 ± 1.44 h versus 2.54 ± 0.21 h), while RA extract pretreatment lengthened *T*_1/2_ in ABCB1 3435CC carriers and abolished such genotype-related difference.	[42]

Grapefruit juice	Ebastine	MDR1	C3435T	The grapefruit juice-induced inhibition of its transport/formation (mean fold-decrease ± SD, 1.5 ± 0.8, 1.1 ± 0.9, and 0.9 ± 0.4) for CC, CT, and TT carriers, respectively.	[43]

Grapefruit juice	Pitavastatin	SLCO1B1	388A>G	Grapefruit juice increased the AUC_0–48 h_ of pitavastatin acid by 14%; SLCO1B1 ^*^1b/^*^1b haplotype (388GG-521TT) had 47% and 44% higher pitavastatin acid exposure than SLCO1B1 ^*^1a carriers (388AA/AG-521TT).	[44]

St. John's wort	Talinolol	MDR1	1236C>T, 2677G>T/A 3435C>T	Subjects harbouring the ABCB1 haplotype comprising 1236C>T, 2677G>T/A, and 3435C>T polymorphisms had lower intestinal MDR1 mRNA levels and showed an attenuated inductive response to St. John's wort as assessed by talinolol disposition.	[45]

St. John's wort	Repaglinide	SLCO1B1	c.521T>C	SLCO1B1 c.521TT genotype subjects presented a trend for lower mean concentrations and AUCs of insulin than c.521TC and CC genotypes subjects, but this trend did not reach statistical significance.	[46]

## References

[1] http://nccam.nih.gov/about.

[2] Eisenberg D. M., Kessler R. C., Foster C., Norlock F. E., Calkins D. R., Delbanco T. L. (1993). Unconventional medicine in the United States: prevalence, costs, and patterns of use. *The New England Journal of Medicine*.

[3] Hori S., Mihaylov I., Vasconcelos J. C., McCoubrie M. (2008). Patterns of complementary and alternative medicine use amongst outpatients in Tokyo, Japan. *BMC Complementary and Alternative Medicine*.

[4] Teng L., Jin K., He K. (2010). Use of complementary and alternative medicine by cancer patients at zhejiang university teaching hospital zhuji hospital, China. *African Journal of Traditional, Complementary and Alternative Medicines*.

[5] Seo H.-J., Baek S.-M., Kim S. G., Kim T.-H., Choi S. M. (2013). Prevalence of complementary and alternative medicine use in a community-based population in South Korea: a systematic review. *Complementary Therapies in Medicine*.

[6] Nakao M., Muramoto Y., Hisadome M. (2007). The effect of Shoseiryuto, a traditional Japanese medicine, on cytochrome P450s, N-acetyltransferase 2 and xanthine oxidase, in extensive or intermediate metabolizers of CYP2D6. *European Journal of Clinical Pharmacology*.

[7] Chan E., Tan M., Xin J., Sudarsanam S., Johnson D. E. (2010). Interactions between traditional Chinese medicines and Western therapeutics. *Current Opinion in Drug Discovery & Development*.

[8] Asdaq S. M., Inamdar M. N. (2010). Potential of garlic and its active constituent, S-allyl cysteine, as antihypertensive and cardioprotective in presence of captopril. *Phytomedicine*.

[9] Gharagozloo M., Moayedi B., Zakerinia M. (2009). Combined therapy of silymarin and desferrioxamine in patients with *β*-thalassemia major: a randomized double-blind clinical trial. *Fundamental & Clinical Pharmacology*.

[10] Tomlinson B., Hu M., Lee V. W. Y. (2008). In vivo assessment of herb-drug interactions: possible utility of a pharmacogenetic approach?. *Molecular Nutrition and Food Research*.

[11] Chen X.-W., Sneed K. B., Pan S.-Y. (2012). Herb-drug interactions and mechanistic and clinical considerations. *Current Drug Metabolism*.

[12] Zhang Z., Ge B., Zhou L., Lam T.-N., Zuo Z. (2014). Induction of liver cytochrome P450s by Danshen-Gegen formula is the leading cause for its pharmacokinetic interactions with warfarin. *Journal of Ethnopharmacology*.

[13] van Strater A. C. P., Bogers J. P. A. M. (2012). Interaction of St John's wort (Hypericum perforatum) with clozapine. *International Clinical Psychopharmacology*.

[14] Naccarato M., Yoong D., Gough K. (2012). A potential drug-herbal interaction between *Ginkgo biloba* and efavirenz. *Journal of the International Association of Physicians in AIDS Care*.

[15] Tian X., Cheng Z.-Y., Jin H., Gao J., Qiao H.-L. (2013). Inhibitory effects of baicalin on the expression and activity of CYP3A induce the pharmacokinetic changes of midazolam in rats. *Evidence-Based Complementary and Alternative Medicine*.

[16] Shao J., Jia L. (2013). Potential serious interactions between nutraceutical ginseng and warfarin in patients with ischemic stroke. *Trends in Pharmacological Sciences*.

[17] Li X., Hu J., Wang B. (2014). Inhibitory effects of herbal constituents on P-glycoprotein in vitro and in vivo: herb-drug interactions mediated via P-gp. *Toxicology and Applied Pharmacology*.

[18] Li L., Hu H., Xu S., Zhou Q., Zeng S. (2012). Roles of UDP-glucuronosyltransferases in phytochemical metabolism of herbal medicines and the associated herb-drug interactions. *Current Drug Metabolism*.

[19] Brantley S. J., Argikar A. A., Lin Y. S., Nagar S., Paine M. F. (2014). Herb-drug interactions: challenges and opportunities for improved predictions. *Drug Metabolism and Disposition*.

[20] Shi S., Klotz U. (2012). Drug interactions with herbal medicines. *Clinical Pharmacokinetics*.

[21] Han S.-Y., Zhao H.-Y., Zhou N., Zhou F., Li P.-P. (2014). *Marsdenia tenacissima* extract inhibits gefitinib metabolism *in vitro* by interfering with human hepatic CYP3A4 and CYP2D6 enzymes. *Journal of Ethnopharmacology*.

[22] Song M., Hong M., Lee M. Y. (2013). Selective inhibition of the cytochrome P450 isoform by hyperoside and its potent inhibition of CYP2D6. *Food and Chemical Toxicology*.

[23] Fan L., Wang J.-C., Jiang F. (2009). Induction of cytochrome P450 2B6 activity by the herbal medicine baicalin as measured by bupropion hydroxylation. *European Journal of Clinical Pharmacology*.

[24] Gorski J. C., Huang S.-M., Pinto A. (2004). The effect of echinacea (*Echinacea purpurea* root) on cytochrome P450 activity in vivo. *Clinical Pharmacology and Therapeutics*.

[25] Yin O. Q. P., Tomlinson B., Waye M. M. Y., Chow A. H. L., Chow M. S. S. (2004). Pharmacogenetics and herb-drug interactions: experience with Ginkgo biloba and omeprazole. *Pharmacogenetics*.

[26] Uno T., Yasui-Furukori N., Takahata T., Sugawara K., Tateishi T. (2005). Lack of significant effect of grapefruit juice on the pharmacokinetics of lansoprazole and its metabolites in subjects with different CYP2C19 genotypes. *The Journal of Clinical Pharmacology*.

[27] Miura M., Kagaya H., Tada H. (2006). Intestinal CYP3A4 is not involved in the enantioselective disposition of lansoprazole. *Xenobiotica*.

[28] Chen Y., Ouyang D.-S., Kang Z. (2012). Effect of a traditional Chinese medicine Liu Wei Di Huang Wan on the activities of CYP2C19, CYP2D6 and CYP3A4 in healthy volunteers. *Xenobiotica*.

[29] Han Y., Guo D., Chen Y., Chen Y., Tan Z.-R., Zhou H.-H. (2009). Effect of silymarin on the pharmacokinetics of losartan and its active metabolite E-3174 in healthy Chinese volunteers. *European Journal of Clinical Pharmacology*.

[30] Wang X.-D., Li J.-L., Su Q.-B. (2009). Impact of the haplotypes of the human pregnane X receptor gene on the basal and St John's wort-induced activity of cytochrome P450 3A4 enzyme. *British Journal of Clinical Pharmacology*.

[31] Xu H., Williams K. M., Liauw W. S., Murray M., Day R. O., McLachlan A. J. (2008). Effects of St John's wort and CYP2C9 genotype on the pharmacokinetics and pharmacodynamics of gliclazide. *British Journal of Pharmacology*.

[32] Rengelshausen J., Banfield M., Riedel K.-D. (2005). Opposite effects of short-term and long-term St John's wort intake on voriconazole pharmacokinetics. *Clinical Pharmacology and Therapeutics*.

[33] Wang L.-S., Zhou G., Zhu B. (2004). St John's wort induces both cytochrome P450 3A4-catalyzed sulfoxidation and 2C19-dependent hydroxylation of omeprazole. *Clinical Pharmacology and Therapeutics*.

[34] Wang L.-S., Zhu B., El-Aty A. M. A. (2004). The influence of St. John's wort on CYP2C19 activity with respect to genotype. *Journal of Clinical Pharmacology*.

[35] Li X., Lian F.-M., Guo D. (2013). The rs1142345 in TPMT affects the therapeutic effect of traditional hypoglycemic herbs in prediabetes. *Evidence-Based Complementary and Alternative Medicine*.

[36] Fan L., Wang G., Wang L.-S. (2007). Herbal medicine *Yin Zhi Huang* induces CYP3A4-mediated sulfoxidation and CYP2C19-dependent hydroxylation of omeprazole. *Acta Pharmacologica Sinica*.

[37] Imanaga J., Kotegawa T., Imai H. (2011). The effects of the SLCO2B1 c.1457C > T polymorphism and apple juice on the pharmacokinetics of fexofenadine and midazolam in humans. *Pharmacogenetics and Genomics*.

[38] Fan L., Zhang W., Guo D. (2008). The effect of herbal medicine baicalin on pharmacokinetics of rosuvastatin, substrate of organic anion-transporting polypeptide 1B1. *Clinical Pharmacology and Therapeutics*.

[39] Hu Y., Ehli E. A., Hudziak J. J., Davies G. E. (2012). Berberine and evodiamine influence serotonin transporter (5-HTT) expression via the 5-HTT-linked polymorphic region. *Pharmacogenomics Journal*.

[40] Mougey E. B., Lang J. E., Wen X., Lima J. J. (2011). Effect of citrus juice and SLCO2B1 genotype on the pharmacokinetics of montelukast. *Journal of Clinical Pharmacology*.

[41] Kim J.-H., Kim S. R., Song I.-S. (2011). Different transport activity of human triallelic MDR1 893Ala/Ser/Thr variant and its association with herb extracts. *Phytotherapy Research*.

[42] Zhou Q., Ye Z., Ruan Z., Zeng S. (2013). Investigation on modulation of human *P*-gp by multiple doses of Radix Astragali extract granules using fexofenadine as a phenotyping probe. *Journal of Ethnopharmacology*.

[43] Gervasini G., Vizcaino S., Carrillo J. A., Caballero M. J., Benitez J. (2006). The effect of *CYP2J2, CYP3A4, CYP3A5* and the *MDR1*
^C3435T^ polymorphisms and gender on the urinary excretion of the metabolites of the H_1_-receptor antihistamine ebastine: a pilot study. *British Journal of Clinical Pharmacology*.

[44] Hu M., Mak V. W. L., Yin O. Q. P., Chu T. T. W., Tomlinson B. (2013). Effects of grapefruit juice and SLCO1B1 388A>G polymorphism on the pharmacokinetics of pitavastatin. *Drug Metabolism and Pharmacokinetics*.

[45] Schwarz U. I., Hanso H., Oertel R. (2007). Induction of intestinal P-glycoprotein by St John's wort reduces the oral bioavailability of talinolol. *Clinical Pharmacology and Therapeutics*.

[46] Fan L., Zhou G., Guo D. (2011). The pregnane X receptor agonist st johns wort has no effects on the pharmacokinetics and pharmacodynamics of repaglinide. *Clinical Pharmacokinetics*.

[47] Wang L., McLeod H. L., Weinshilboum R. M. (2011). Genomics and drug response. *The New England Journal of Medicine*.

[48] Hu M., Wang D. Q., Xiao Y. J., Mak V. W. L., Tomlinson B. (2012). Herb-drug interactions: methods to identify potential influence of genetic variations in genes encoding drug metabolizing enzymes and drug transporters. *Current Pharmaceutical Biotechnology*.

[49] Hu M., Fan L., Zhou H.-H., Tomlinson B. (2012). Theranostics meets traditional Chinese medicine: rational prediction of drug-herb interactions. *Expert Review of Molecular Diagnostics*.

[50] Mohammed Abdul M. I., Jiang X., Williams K. M. (2008). Pharmacodynamic interaction of warfarin with cranberry but not with garlic in healthy subjects. *British Journal of Pharmacology*.

